# S-glutathionylation reactions in mitochondrial function and disease

**DOI:** 10.3389/fcell.2014.00068

**Published:** 2014-11-17

**Authors:** Ryan J. Mailloux, William G. Willmore

**Affiliations:** ^1^Department of Biology, Faculty of Sciences, University of OttawaOttawa, ON, Canada; ^2^Institute of Biochemistry, Carleton UniversityOttawa, ON, Canada

**Keywords:** glutathionylation, mitochondria, oxidative phosphorylation, glutaredoxins, reactive oxygen species (ROS)

## Abstract

Mitochondria are highly efficient energy-transforming organelles that convert energy stored in nutrients into ATP. The production of ATP by mitochondria is dependent on oxidation of nutrients and coupling of exergonic electron transfer reactions to the genesis of transmembrane electrochemical potential of protons. Electrons can also prematurely “spin-off” from prosthetic groups in Krebs cycle enzymes and respiratory complexes and univalently reduce di-oxygen to generate reactive oxygen species (ROS) superoxide (O_2_•^−^) and hydrogen peroxide (H_2_O_2_), important signaling molecules that can be toxic at high concentrations. Production of ATP and ROS are intimately linked by the respiratory chain and the genesis of one or the other inherently depends on the metabolic state of mitochondria. Various control mechanisms converge on mitochondria to adjust ATP and ROS output in response to changing cellular demands. One control mechanism that has gained a high amount of attention recently is S-glutathionylation, a redox sensitive covalent modification that involves formation of a disulfide bridge between glutathione and an available protein cysteine thiol. A number of S-glutathionylation targets have been identified in mitochondria. It has also been established that S-glutathionylation reactions in mitochondria are mediated by the thiol oxidoreductase glutaredoxin-2 (Grx2). In the following review, emerging knowledge on S-glutathionylation reactions and its importance in modulating mitochondrial ATP and ROS production will be discussed. Major focus will be placed on Complex I of the respiratory chain since (1) it is a target for reversible S-glutathionylation by Grx2 and (2) deregulation of Complex I S-glutathionylation is associated with development of various disease states particularly heart disease. Other mitochondrial enzymes and how their S-glutathionylation profile is affected in different disease states will also be discussed.

## Introduction

**Figure 1:**
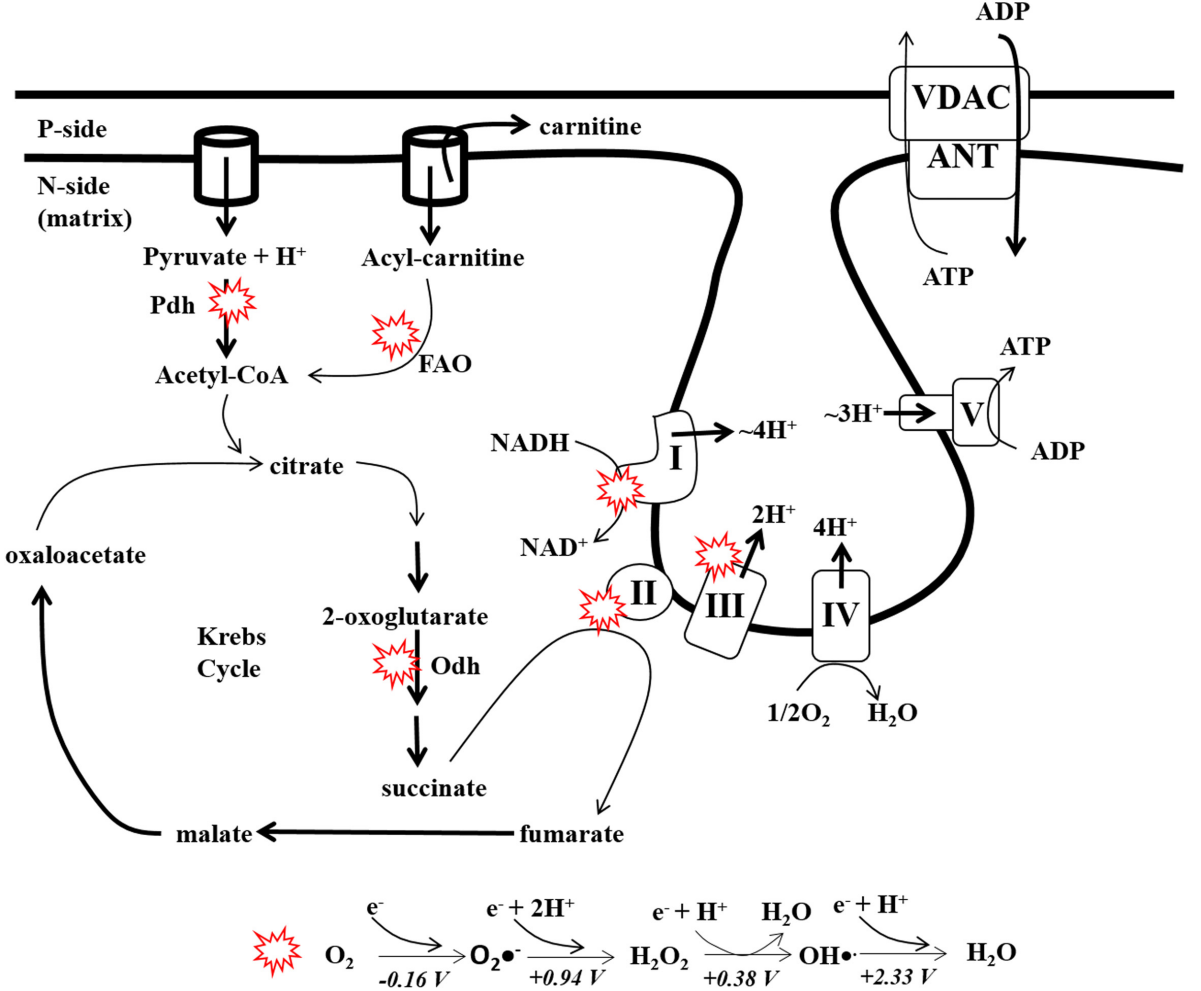
**Production of ATP and ROS by the electron transport chain**. Nutrients in the form of glucose, fatty acids, or amino acids are converted into the Krebs cycle intermediates and then oxidized by 8 different enzymes. During carbon oxidation electrons are extracted and ferried through the respiratory complexes to drive ATP production (OXPHOS). Electrons from the Krebs cycle enter the respiratory chain via NADH at Complex I or succinate at Complex II. Electron flow to the terminal acceptor di-oxygen which creates the transmembrane electrochemical potential of protons which drives ATP production by Complex V. *Red circles*: represent major sites for ROS production in mitochondria (Univalent reduction of di-oxygen (O_2_) to H_2_O. The standard redox potentials for each univalent reduction of O_2_, O_2_•^−^, H_2_O_2_, and OH•^.^ are indicated in the diagram). Pdh; pyruvate dehydrogenase, Odh; 2-oxoglutarate dehydrogenase, I, Complex I; II, Complex II; III, Complex III; IV, Complex IV; V, Complex V; ANT, adenine nucleotide translocase; VDAC, voltage-dependent anion channel.

The degree of biological complexity is inherently related to energy flux (Lane and Martin, 2010; Wallace, 2010). In aerobic eukaryotes most energy transfer reactions take place in mitochondria, double membrane organelles that transform energy stored in nutrients into ATP. Energy conversion in mitochondria involves coupling exergonic electron transfer reactions to the production of ATP, an indispensable form of energy required to perform work in the cell. Aerobic respiration starts when electrons are stripped from nutrients via the concerted action of eight different Krebs cycle enzymes which results in the production of the electron carriers NADH and succinic acid (Figure 1) (Brand and Nicholls, 2011; Mailloux and Harper, 2012; Verkhovskaya and Bloch, 2013). Electrons from NADH and succinic acid are then transferred to respiratory Complex I (NADH:ubiquinone oxidoreductase) and Complex II (Succinate dehydrogenase; Sdh) and then systematically passed through a series of prosthetic groups to ubiquinone forming ubiquinol. Other enzymes including electron transfer flavoprotein oxidoreductase (ETF-QO), dihydroorotate dehydrogenase, FAD-linked glycerol-3-phosphate dehydrogenase, proline dehydrogenase, and sulfide:quinone oxidoreductase (SQR) also donate electrons from their cognate substrates to ubiquinone (Mailloux et al., 2013a; Quinlan et al., 2014). The electrons are then passed through prosthetic groups in respiratory Complex III (Ubiquinol:cytochrome C oxidoreductase) and Complex IV (Cytochrome C oxidase; COX) to O_2_ (Figure 1). Electron transfer through the Complexes from NADH to O_2_ is energetically favorable which is coupled to the pumping of protons into the intermembrane space (IMS) from the matrix (matrix) generating a protonmotive force (pmf) which is tapped by Complex V (ATP synthase) for ATP synthesis (Walker, 2013).

Bacteria also harbor enzymes and protein complexes that couple nutrient metabolism to the reduction of O_2_ and production of ATP. Thus, it is important to consider why mitochondria are responsible for the evolution of more complex life and why biological complexity is out of the humble bacterium's grasp. The efficiency of aerobic metabolism in bacteria depends on the ratio of membrane surface area to cell volume (Lane and Martin, 2010; Lane, 2014). As the size of a microbe increases there is a hyperbolic decrease in respiratory and nutrient absorption efficiency (Lane, 2005, 2014). Mitochondria overcome surface area restraints with a highly folded selectively permeable and energy transducing mitochondrial inner membrane (MIM). The inward folding of the MIM creates deep invaginations called cristae enriched in respiratory complexes and Krebs cycle enzymes which amplifies energy transduction. The mitochondrial MIM is ~5–7 nm thick and can generate a transmembrane electrochemical proton potential of ~150–180 mV (Perkins et al., 1997; Gerencser et al., 2012). Thus, the voltage gradient experienced by the MIM in respiring mitochondria is ~300, 000 V cm^−1^. In addition, mammalian cells contain a number of mitochondria which can occupy ~2–40% of the cell volume (Brand, 2014). Based on this it is easy to see why a mitochondrion can conserve far more energy from nutrient oxidation than a microbe.

Mitochondria are highly dynamic organelles that are required to furnish human tissues with ATP. Different tissues have different energy demands which will dictate the number of mitochondria within the tissue as well as its structure, size, density, and ATP output. Various control mechanisms converge on mitochondria to regulate nutrient uptake, ATP output, and ROS production/degradation which is dependent on the energy demands of the tissue. Coarse control mechanisms including mitochondrial fission and fusion, changes in cristae structure, respirasome assembly, protein complex assembly, protein degradation and expression of nuclear and mitochondrial genes play intricate roles in modulating ATP output by mitochondria. Rapid control mechanisms include allosteric interactions and covalent modifications. Deregulation of mitochondrial control mechanisms have been associated with a number of pathologies including heart disease, neurological disorders, obesity, diabetes mellitus, and non-alcoholic fatty liver disorder (Mailloux and Harper, 2011). Protein thiol oxidation reactions, in particular S-glutathionylation, have also been found to play a crucial role in modulating mitochondrial physiology, structure, and bioenergetics (Hurd et al., 2005; Mailloux et al., 2013b; Drose et al., 2014). Here, we will survey the importance of S-glutathionylation reactions in the control of mitochondrial metabolism. Special emphasis will be given to Grx2 and the control of Complex I activity and function and how deregulation of S-glutathionylation cascades can lead to development of disease.

## Mitochondrial oxyradical homeostasis

Di-oxygen has two unpaired electrons in its anti-bonding orbitals with parallel spins and can accept only one electron at a time. Thus, reduction of O_2_ to H_2_O can result in the formation of oxyradical intermediates, specifically superoxide (O_2_•^−^), hydrogen peroxide (H_2_O_2_), and hydroxyl radical (OH•^.^) (Figure 1) (Imlay, 2013). Superoxide is a membrane impermeant weak oxidant and reductant and reacts with very few biological molecules (Imlay, 2003). An important exception are iron-sulfur clusters (Fe-S) which are in high abundance in mitochondria (Chepelev and Willmore, 2011; James et al., 2012). O_2_•^−^ reactivity and diffusion capacity can be increased if it can be protonated to form its conjugated acid, perhydroxyl radical (HO_2_•^−^) (De Grey, 2002). Since protonation/deprotonation equilibrium of O_2_•^−^ has a pKa of ~4.88 most of the O_2_•^−^ will exist in the deprotonated state (~0.3% of O_2_•^−^ exists as HO_2_•^−^) (De Grey, 2002). Hydrogen peroxide is a strong two electron oxidant but also reacts with few biological molecules due to activation energy restraints (Winterbourn, 2013). Importantly, H_2_O_2_ is able to diffuse through membranes via aquaporins and reacts strongly with seleno groups and protein cysteine thiols (-SH) which forms the basis of antioxidant defense and redox signaling (Finkel, 2011; Mailloux et al., 2013a). Hydroxyl radical is by far the most reactive molecule in the group and is able to oxidize a number of different biological macromolecules. Hydroxyl radical has a redox potential of ~2 V and its reactivity is limited by diffusion (Imlay, 2013). The concentration of different ROS species in a cell ranges from H_2_O_2_ > O_2_•^−^ > OH•^.^ with H_2_O_2_ being found in the sub-μM range (10^−7^–10^−9^ M) and O_2_•^−^ in the low to mid pM range (Murphy, 2009; Sies, 2014). Concentrations of OH^.^ are much more difficult to estimate given its rapid reaction kinetics (~10^9^ M^−1^ s^−1^) (Samuni et al., 2002).

H_2_O_2_ and to a lesser extent O_2_•^−^ play intricate roles in mitochondrial signaling to the rest of the cell. However, in large quantities O_2_•^−^ and H_2_O_2_ can be harmful. Overproduction of O_2_•^−^ can lead to the indiscriminant disassembly of Fe-S clusters, in particular in aconitase (Acn) and sometimes Sdh, Fumarase (Fum), and Complex I (James et al., 2012). Superoxide can also combine with nitric oxide to generate peroxynitrite (ONOO^−^) which rapidly oxidizes biological macromolecules (Beckman and Koppenol, 1996). Overproduction of mitochondrial O_2_•^−^ and the disassembly of Fe-S clusters can disable nutrient oxidation and ATP production which has been implicated in the pathogenesis of non-alcoholic fatty liver disease, obesity, diabetes mellitus, and several neurological disorders (James et al., 2012). Hydrogen peroxide can non-selectively oxidize protein amino acids in particular cysteine and methionine rendering proteins inactive and/or amenable to electrophilic modification (Kaspar et al., 2009). Hydrogen peroxide can also participate in Haber-Weiss reactions with O_2_•^−^ to produce OH^.^ (Khan and Kasha, 1994). Over production of either O_2_•^−^ and H_2_O_2_ has been associated with mitochondrial DNA, protein, and lipid damage, metabolic dysfunction and oxidative stress (Berlett and Stadtman, 1997). In addition, over production of ROS due to mitochondrial dysfunction has been linked to a number of different pathologies including neurological diseases, heart disease, obesity, and diabetes mellitus. ROS species are dangerous but indispensable to mitochondrial function and signaling. Thus, it is crucial for mitochondria to strike a balance between ROS production and degradation which requires various antioxidant systems and mechanisms that control ROS production.

Mitochondria can be a major source of reactive oxygen species (ROS) in the aerobic cell (Brown and Borutaite, 2012). It is estimated that ~0.2–0.5% of the O_2_ consumed by mitochondria is converted to ROS (Chance et al., 1979) however; the actual amount of ROS generated by mitochondria depends on its metabolic poise, redox state, O_2_ saturation, and concentration of the redox active enzyme that can generate ROS. Accessibility of an enzymatic ROS-generating site to di-oxygen is also a key determinant for rate of ROS production (Klinman, 2007). Superoxide is the proximal ROS species generated by mitochondria and can be produced by a number of different enzymes and respiratory complexes in the Krebs cycle and ETC (Figure 1) (Mailloux et al., 2013a). Complex I and III are most often, if always, viewed as the major sites for O_2_•^−^ production. While the former generates O_2_•^−^ on the matrix side of the mitochondria only the latter can generate O_2_•^−^ on either intermembrane space side or matrix side of the MIM (Turrens and Boveris, 1980). Experiments utilizing different substrates and inhibitors have established that flavin mononucleotide (FMN) prosthetic group is the major site for O_2_•^−^ genesis by Complex I (Kudin et al., 2004; Lambert and Brand, 2004). Another potential site for O_2_•^−^ production by Complex I is the Q-binding site which exhibits high sensitivity to Δ pH (Treberg et al., 2011). By utilizing different substrate combinations and inhibitors like Antimycin A, stigmatellin, myxothiazol, and a Rieske Fe-S knockout model, it was established that the Q_o_ site of Complex III can also generate large amounts of O_2_•^−^ (Brunelle et al., 2005; Miwa and Brand, 2005). In addition, Complex III releases most of its O_2_•^−^ into the intermembrane space where it can participate in redox signaling however; this may only occur under certain circumstances such as hypoxia (Guzy et al., 2005). Note that mitochondrial ROS production measurements are usually conducted at ambient oxygen ([O_2_]_atmosphere_ ~200 μM at 37°C) and in normal physiological conditions the concentration of di-oxygen experienced by mitochondria is ~3–30 μM (Turrens, 2003). However, considering the low concentration of O_2_•^−^ in mitochondria and standard reduction potential of O_2_•^−^ (−0.160 V, pH 7.0), its production will always be favorable even if [O_2_] were as low as 1 μM (Murphy, 2009).

Since Complex I and III are often heralded as the chief sites of mitochondrial ROS production, other sites including 2-oxoglutarate dehydrogenase (Odh), branched chain amino acid dehydrogenase (Bckdh), pyruvate dehydrogenase (Pdh), Sdh, dihydroorotate dehydrogenase, FAD-linked glycerol-3-phosphate dehydrogenase, proline dehydrogenase, and electron transfer flavoprotein ubiquinone:oxidoreductase are often overlooked (Murphy, 2012a). Intriguingly, recent reports have actually shown that depending on which substrate is being metabolized, the redox state of NADH/NAD, and whether or not electron transfer inhibitors are present, the chief site of mitochondrial ROS can vary drastically from one enzyme to the next. Blockage of the ETC or high substrate concentration can induce an increase in ROS production by Complex I and III. For instance, succinate at a final concentration of 5 mM can induce a substantial increase in O_2_•^−^ production from the FMN site of Complex I (Pryde and Hirst, 2011). Considering that the physiological concentration of succinate is in the μM range it does not seem that reverse electron transfer from Complex II to Complex I is a significant physiological source of mitochondrial ROS (Mailloux and Harper, 2011). In addition, pyruvate and malate/glutatamate, which generate Complex I substrate NADH, produce very little O_2_•^−^ when added to reaction chambers (Muller et al., 2008). The amount of O_2_•^−^ produced by pyruvate and malate/glutamate metabolism can be amplified by rotenone, a Complex I inhibitor (Muller et al., 2008). What is important to note here is that addition of rotenone increases the amount of NADH which can induce a substantial increase in O_2_•^−^/H_2_O_2_ production by Odh and Pdh (Starkov et al., 2004; Fisher-Wellman et al., 2013). In one particular study, Quinlan et al. provided conclusive evidence that Odh and Pdh, rather than the Complexes, serve as the major sites for O_2_•^−^/H_2_O_2_ production in mitochondria (Quinlan et al., 2014). In addition the authors contend that based on their results mitochondrial O_2_•^−^/H_2_O_2_ production can be mistakenly associated with Complex I rather than Odh and Pdh (Quinlan et al., 2014). Both Odh and Pdh are composed of three enzymes; 2-oxoglutarate or pyruvate dehydrogenase (E_1_), dihydrolipoyl succinyltransferase or dihydrolipoyl acetyltransferase (E_2_), and dihydrolipoyl dehydrogenase (E_3_) (McLain et al., 2011). The E_2_ subunit contains a lipoic acid residue which is extremely important for catalysis of substrate oxidation and decarboxylation but is also highly amenable to redox modifications. The E_3_ subunit harbors an FAD group which can readily participate in O_2_•^−^/H_2_O_2_ especially if NADH is high (Ambrus et al., 2009). It has been known for some time that flavins can generate O_2_•^−^/H_2_O_2_ in O_2_ saturated aqueous solutions and that Odh and Pdh can generate ROS (Starkov et al., 2004). However, the recent work by Quinlan et al. really brings into question whether or not Complex I or Odh and to a lesser extent Pdh are the major ROS producing sites in mitochondria (Quinlan et al., 2014). This study also indicates that Odh and Pdh rather than the ETC Complexes may serve as the central hub for ROS signaling from mitochondria to the rest of the cell and thus may be an important pharmacological target for treatment of various diseases. For instance, recent work published by Stuart et al. showed that lipoic acid analog CPI-613 selectively kills cancer cells by amplifying ROS production from the E_3_ subunit of Odh (Stuart et al., 2014). Thus, Odh and Pdh need to be seriously considered as major contributors to mitochondrial O_2_•^−^/H_2_O_2_ genesis for ROS signaling and mitochondrial dysfunction.

### Mitochondrial transport of glutathione and ROS degradation

Glutathione concentrations in the mitochondrial matrix are equivalent to that found in the cytosol (Ribas et al., 2014). Typically, glutathione within the mitochondria is maintained at a high level; between 5 and 10 mM, and comprises ~10–15% of the total cellular glutathione in the liver (Jocelyn and Kamminga, 1974) and ~30% in the kidney (Schnellmann, 1991). Since the production of glutathione occurs exclusively in the cytosol and mitochondria lack the enzymes of glutathione synthesis and thus all mitochondrial glutathione must be imported. Potential candidates for glutathione importers include the 2-oxoglutarate carrier (OGC) and the dicarboxylate carrier (DIC) in the liver and kidney and the tricarboxylate carrier (TTC) in the brain (Mari et al., 2013). Interestingly, a role for UCP2 in the transport of mitochondrial glutathione has been reported in neurons (de Bilbao et al., 2004). This study suggests that the transport of protons back into the matrix by UCP2 may favor the movement of glutathione.

Mitochondria contain a myriad of antioxidant defense enzymes and low molecular weight molecules that degrade both O_2_•^−^ and H_2_O_2_ with exquisite efficiency (Mailloux et al., 2013a). The antioxidant defense system in mitochondria has been reviewed extensively (Murphy, 2012b; Mailloux et al., 2013a). Of importance for our purposes in here is that mitochondrial glutathione plays a number of roles as an antioxidant including the direct sequestration of H_2_O_2_, detoxification of lipid hydroperoxides, and S-glutathionylation of proteins to protect from oxidative damage. Importantly, GSH itself does not spontaneously sequester hydroperoxides since the reaction kinetics are far too slow (30 M^−1^ s^−1^) (Berndt et al., 2014a). Rather, the capacity of glutathione to detoxify H_2_O_2_ and lipid hydroperoxides or participate in covalent modification of protein cysteine thiols depends on various enzymes that specifically bind glutathione to catalyze redox reactions (Flohe, 2013; Berndt et al., 2014a). Indeed, H_2_O_2_ and lipid hydroperoxide detoxification are enzymatically-mediated by glutathione peroxidase (Gpx) 1 and Gpx4. Gpx1 is the matrix soluble isoform while Gpx4 is bound to the MIM (Mailloux et al., 2013a). In addition, Gpx1 preferentially scavenges H_2_O_2_ and Gpx4 is mostly involved in the elimination of lipid hydroperoxides. The scavenging of H_2_O_2_ via the glutathione system requires two GSH molecules. Interaction with H_2_O_2_ generates GSSG which is then reduced back to GSH by NADPH and glutathione reductase (Flohe, 2013). Mitochondria contain a number of NADPH generating enzymes which are required to funnel additional reductive power to antioxidant systems (Mailloux et al., 2013a).

## S-glutathionylation reactions in mitochondria

### Non-enzymatic protein S-glutathionylation

Protein cysteine SH groups can be subjected to a range of different redox modifications which are dependent on H_2_O_2_ levels, GSSG concentration, presence of H_2_S, availability of nitric oxide, proximity of neighboring SH groups (for inter or intramolecular disulfide formation), proximity of amides or sulfenic acid residues (Goubern et al., 2007; Grek et al., 2013; Mailloux et al., 2013b). Since we are focusing on S-glutathionylation reactions we will not discuss the other protein cysteine SH redox modifications. However, we encourage the reader to consult the following comprehensive reviews for detailed information on all other known redox modifications (Murphy, 2012b; Grek et al., 2013; Mailloux et al., 2013b). Protein S-glutathionylation can proceed either non-enzymatically or enzymatically. Non-enzymatic S-glutathionylation reactions are classically viewed as occurring when the 2GSH/GSSG ratio is ~1, e.g., during oxidative stress (Ziegler, 1985; Gallogly and Mieyal, 2007). However, it has now been established that glutathione S-transferases catalyze protein S-glutathionylation (Klaus et al., 2013). Non-enzymatic PSSG formation can proceed as follows; (1) simple disulfide exchange with GSSG granted that GSSG is sufficiently concentrated (2GSH/GSSG is ~1), (2) S^−^ is oxidized by H_2_O_2_ generating a sulfenic acid (SOH) which, due to its very low pKa, can ionize and rapidly react with reduced glutathione (GSH), and (3) S^−^ forms a thiyl radical which then interacts with GSH forming a thiyl radical glutathionyl intermediate which then interacts with O_2_ to form PSSG (Mailloux et al., 2013a). Spontaneous S-glutathionylation can play important roles such as protecting SH groups from irreversible oxidation by H_2_O_2_ during oxidative stress (Nulton-Persson et al., 2003). However, it has also been suggested that spontaneous PSSG formation could also occur under normal conditions. This would be dependent on the state of redox microenvironments. For example due to its extreme folding, the mitochondrial MIM creates mitochondrial sub-compartments in the matrix and intermembrane space which can generate very distinct microenvironments with different redox signatures (Drose et al., 2014). This would mean that mitochondria could create various redox gradients under normal conditions which would render proteins more amenable toward spontaneous S-glutathionylation under normal conditions. The capacity of a protein to undergo spontaneous S-glutathionylation would also depend on its reactivity toward either GSSG or H_2_O_2_. It has been reported that the K_ox_ for a typical protein is ~1 (when 2GSH/GSSG is ~1) (Gilbert, 1995). However, some proteins like c-Jun have a K_ox_ of ~13 and therefore 50% of c-Jun is S-glutathionylated when 2GSH/GSSG is ~13 (Klatt et al., 1999). This could also be the case for some mitochondrial proteins like Complex I and Odh which seem to be quite sensitive to S-glutathionylation (Beer et al., 2004; Applegate et al., 2008). In addition, a recent report has established that proteins and enzymes have S-glutathionylation motifs which are rich in positively charged amino acids surrounding a modifiable cysteine (Chen et al., 2014). Thus, although non-enzymatic or spontaneous S-glutathionylation may occur in response to oxidative stress, this type of S-glutathionylation could also proceed in normal cells considering that the 2GSH/GSSG pool is dynamic and that redox gradients can be created in various mitochondrial compartments and subcompartments.

### Enzymatically driven protein S-glutathionylation reactions

It has long been known that cytosolic thiol oxidoreductase glutaredoxin-1 (Grx1) catalyzes the deglutathionylation of cytosolic proteins (Mannervik and Axelsson, 1980). *In vitro* Grx1 is also able to deglutathionylate purified mitochondrial matrix proteins or enzymes in permeabilized mitochondria (Gallogly et al., 2009; Stroher and Millar, 2012). Grx1 is a small heat-stable enzyme and a member of the thioredoxin (Trx) superfamily (Stroher and Millar, 2012). Grx1 harbors has a thioredoxin fold (4 β-sheets surrounded by α-helices) and contains a catalytic CXXC motif located on the loop region at the end of the first α-helix (Stroher and Millar, 2012). Grx1 catalyzes deglutathionylation of target proteins by nucleophilic displacement which has been very well characterized and reviewed extensively elsewhere (Holmgren, 1988; Gallogly et al., 2009). The rate of Grx1-mediated protein deglutathionylation varies substantially between 1.75 × 10^5^ and 4.0 × 10^2^ M^−1^s^−1^ which is heavily dictated by the pKa of the target thiol (e.g., the lower the pKa the faster the rate) and accessibility (Jensen et al., 2014). The mitochondrial matrix contains a Grx1 isozyme, Grx2 (Gladyshev et al., 2001; Lundberg et al., 2001). Grx2 was identified a little over a decade ago, shares ~34% sequence identity with Grx1, and is highly concentrated (~1 μM in comparison to Grx1 which is ~0.1 μM) in mitochondria (Gallogly et al., 2008). Grx2 is also a dithiol oxidoreductase and uses a similar catalytic mechanism to Grx1 for the deglutathionylation of target proteins. An exception here is that the Grx2-SSG intermediate can also be reduced by thioredoxin reductase (TrxR) in an NADPH-dependent fashion (Johansson et al., 2004). It has also been reported that Grx2 can reduce mitochondrial Trx2 (Zhang et al., 2014). These observations are intriguing since Trx reductase (TrxR) is the only enzyme known to reduce Trx2. TrxR is also very sensitive to deactivation by electrophiles like 4-hydroxy-2-nonenal (Zhang et al., 2014). Thus, Grx2 is able to maintain Trx2 activity when TrxR is deactivated under oxidative stress. Notably, Grx2 is not sensitive to deactivation by electrophilic or oxidative stress. Grx2 has also been reported to catalyze the reduction of intra and intermolecular thiol disulfide bridges. This is intriguing considering that Grx2 deglutathionylase activity operates via a monothiol mechanism which requires only the N-terminal active site cysteine. Reduction of disulfides requires both N-terminal and C-terminal cysteines which has been reviewed in Lillig and Berndt (2013).

Grx2 is modulated by the assembly of 2Fe-2S cluster which maintains Grx2 as an inactive dimer (Lillig et al., 2005). Disassembly of the 2Fe-2S cluster leads to the release of two activate Grx2 monomers which can then deglutathionylate protein targets in mitochondria. It has been suggested that the 2Fe-2S cluster serves as a sensor for oxidative stress and fluctuations in redox environment in mitochondria (Lillig et al., 2005). A burst in ROS production in mitochondria, most likely O^−•^_2_, would lead to oxidation of the glutathione pool and the subsequent S-glutathionylation of various proteins. Active Grx2 would then be required to restore the S-glutathionylated proteome in mitochondria. Grx2 is then subsequently deactivated by 2Fe-2S cluster reassembly (Qi and Cowan, 2011). In a recent study, Gao et al. were able to activate holo-Grx2 using a xanthine/xanthine oxidase system indicating O^−•^_2_ may be responsible for 2Fe-2S cluster disassembly (Gao et al., 2013). It is important to point out that in the same study the authors failed to activate Grx2 in mitochondria incubated in 20 mM glutamate and 7.5 μM rotenone (Gao et al., 2013). However, it should be noted that O^−•^_2_ production was not measured and other potential substrates known to generate high amounts of O^−•^_2_, like succinate in conjunction with antimycin A, were never tested. Grx1 and Grx2 also have glutathionylase activity. For Grx1 this is thought to occur via the stabilization of a glutathionyl radical which then ultimately results in its transfer to a target protein (Gallogly et al., 2008). A similar mechanism may exist for Grx2 however; Grx2 glutathionylase activity seems to be activated by the redox state of the 2GSH/GSSG pair. A more oxidized glutathione pool activates the glutathionylase activity of Grx2 whereas a reduced pool has the opposite effect (Beer et al., 2004). This has been effectively demonstrated for Complex I in bovine and mouse heart mitochondria (Beer et al., 2004) How an oxidized glutathione pool activates the glutathionylase activity of Grx2 remains unclear however; our group has suggested a potential mechanism in a previous publication (Mailloux et al., 2013b). Fe-S cluster coordination by Grx2 also requires 2GSH molecules which are required to stabilize the Grx2 dimer—2Fe2S cluster assembly (Berndt et al., 2007; Lillig and Berndt, 2013). It is possible that oxidation of the GSH pool also results in 2Fe-2S cluster disassembly and activation of Grx2 via an initial liberation of GSH from the complex.

### Grx2 in embryonic development, health, and disease

Cellular redox balance is essential for modulating protein activities in mammalian cells. To this end the thiol oxidoreductase activity of Grx2 has been shown to be indispensable for proper mitochondrial function in various tissues. Selective overexpression of Grx2 in mouse heart protects from doxorubicin induced cardiac injury (Diotte et al., 2009). Grx2 overexpression also protects from ischemia-reperfusion injury, apoptosis, and reduces infarct formation in heart tissue (Gallogly et al., 2010; Wu et al., 2010). Likewise Grx2 knockout induces left ventricular hypertrophy and localized fibrosis in mice (Mailloux et al., 2014). Grx2 overexpression has also been shown to curtail MPP^+^ toxicity in neuroblastoma cells and is upregulated rapidly in midbrain and striatum following MPTP treatment (Karunakaran et al., 2007). A report has also suggested that gene therapy, specifically with thioredoxin superfamily genes (e.g., Grx2), may serve as a novel avenue for treatment of heart disease (Ahsan et al., 2009). Through a series of publications Brautigam et al. and Berndt et al. were able to show that Grx2 plays a critical role in embryonic development. Specifically, using a zebrafish model and various types of cultured cells, it was shown that Grx2 plays a pivotal role in brain development (Brautigam et al., 2011), regulates vascularization via Sirt1 S-glutathionylation (Brautigam et al., 2013), and heart development by regulating neural crest cell migration and survival (Berndt et al., 2014b). Loss of Grx2 curtails these development pathways which severely hampers embryonic development. Collectively, these studies highlight the important function of redox signaling, mediated by Grx2, in embryonic development, cell function, and tissue health.

## Mitochondria are important sites for regulation by S-glutathionylation

S-glutathionylation is required to modulate cytoskeletal dynamics, cell motility, cell division, ion transport, calcium homeostasis, gene expression, epigenetics, glycolysis, energy sensing through AMP kinase, bicarbonate metabolism, and apoptosis (Cooper et al., 2011; Pimentel et al., 2012). Deregulation of reversible S-glutathionylation also has strong implications for health and disease. Like many other pathological conditions that involve deregulated covalent modifications (e.g., phosphorylation or acetylation), altered S-glutathionylation patterns have been linked to various disease states including obesity, diabetes mellitus, cardiovascular disease, inflammation, cancer, and aging (Cooper et al., 2011; Picklo et al., 2013; Sanchez-Gomez et al., 2013; Seo and Lee, 2014). Emerging evidence now indicates that S-glutathionylation reactions also play a central role in modulating mitochondrial function in various organs and tissues. The mitochondrial matrix favors S-glutathionylation reactions (Murphy, 2012b). A review of the literature reveals that a number of mitochondrial proteins involved in a diverse array of processes can be S-glutathionylated (reviewed by Mailloux et al., 2013a,b; Drose et al., 2014). Most of these proteins seem to be S-glutathionylated under oxidative stress and it remains unknown if S-glutathionylation is required to regulate their physiological function(s) (Mailloux et al., 2013a). From the list of 15 proteins involved in metabolism, ROS homeostasis, and apoptosis, only a handful seem to be (1) regulated by reversible S-glutathionylation and (2) display altered S-glutathionylation patterns in various pathologies (Mailloux et al., 2013a). In addition, out of this same list of proteins Complex I of the ETC has been identified as the only *bona fide* target for Grx2 action (Mailloux et al., 2013a). Recent reports have also linked Grx2 function to the regulation of UCP3 (Mailloux et al., 2013c). However, knockout of Grx2 in mice results in S-glutathionylation of a number of proteins at various molecular weights in liver and heart mitochondria suggesting that Grx2 may have a multitude of targets (Mailloux et al., 2013c, 2014).

### Aconitase (Acn)

Acn is the second enzyme of the Krebs cycle and catalyzes the isomerization of citrate to isocitrate through a *cis*-aconitate intermediate. Catalysis is dependent on a cubane [4Fe-4S]^2+^ cluster which is required to facilitate the elimination of the hydroxyl group from C_3_ position of citrate (Gardner, 1997). Specifically the solvent exposed Fe_a_ group in the cluster is required for coordination of the OH group on the C_3_ prompting its elimination and the genesis of a *cis*-aconitate intermediate (Lauble and Stout, 1995). The solvent exposed Fe-S cluster in Acn also makes it highly amenable for deactivation by O_2_•^−^. Inactivation is caused by oxidation of the cluster followed by release of Fe_a_ (which is required to coordinate OH on C_3_ position of citrate) and cluster disassembly (Gardner, 1997). Second-order rate constants for this reaction are ~10^7^ M^−1^s^−1^ (Flint et al., 1993). However, it should be noted that [4Fe-4S]^2+^ clusters can also be disassembled by ONOO^−^ (Kennedy et al., 1997). Nevertheless, Acn deactivation during oxidative stress is associated with various diseases including obesity, cardiovascular diseases, and neurological disorders (Wlodek and Gonzales, 2003; Goncalves et al., 2010).

Recent work has established that Acn is also a key site for modulation by cysteine oxidation reactions (Han et al., 2005). It has been demonstrated that cysteine residues in close proximity to the citrate binding site, specifically Cys^126^ and Cys^385^, can form sulfenic (SOH) and sulfinic (SO_2_H) acids in the presence of H_2_O_2_ (Han et al., 2005). These cysteine residues are also amenable to S-glutathionylation which lowers Acn activity most likely through blocking the active site (Han et al., 2005). S-glutathionylation is also inversely proportional to the concentration of citrate (Han et al., 2005). Thus, Acn S-glutathionylation is dependent on whether or not citrate is available to occupy the active site. It has long been known that citrate or analogs like tricarballylic acid are required to bind and stabilize the [4Fe-4S]^2+^ in Acn during isolation procedures. It is entirely possible that S-glutathionylation of active site cysteine residues may protect Acn from irreversible deactivation in various tissues by preventing Fe-S disassembly by O_2_•^−^ and ONOO^−^ when citrate (or *cis*-aconitate and isocitrate) is unavailable to occupy the active site. In addition, deregulation of S-glutathionylation of Acn may contribute to the pathogenesis of various disorders including obesity and neurological diseases. As noted above Acn is a major target for deactivation by oxidative stress in various pathologies. Thus, disruption of Acn S-glutathionylation could amplify the probability of deactivation and may contribute to development of various diseases.

### Pdh, Odh, and Bckdh

**Figure 2:**
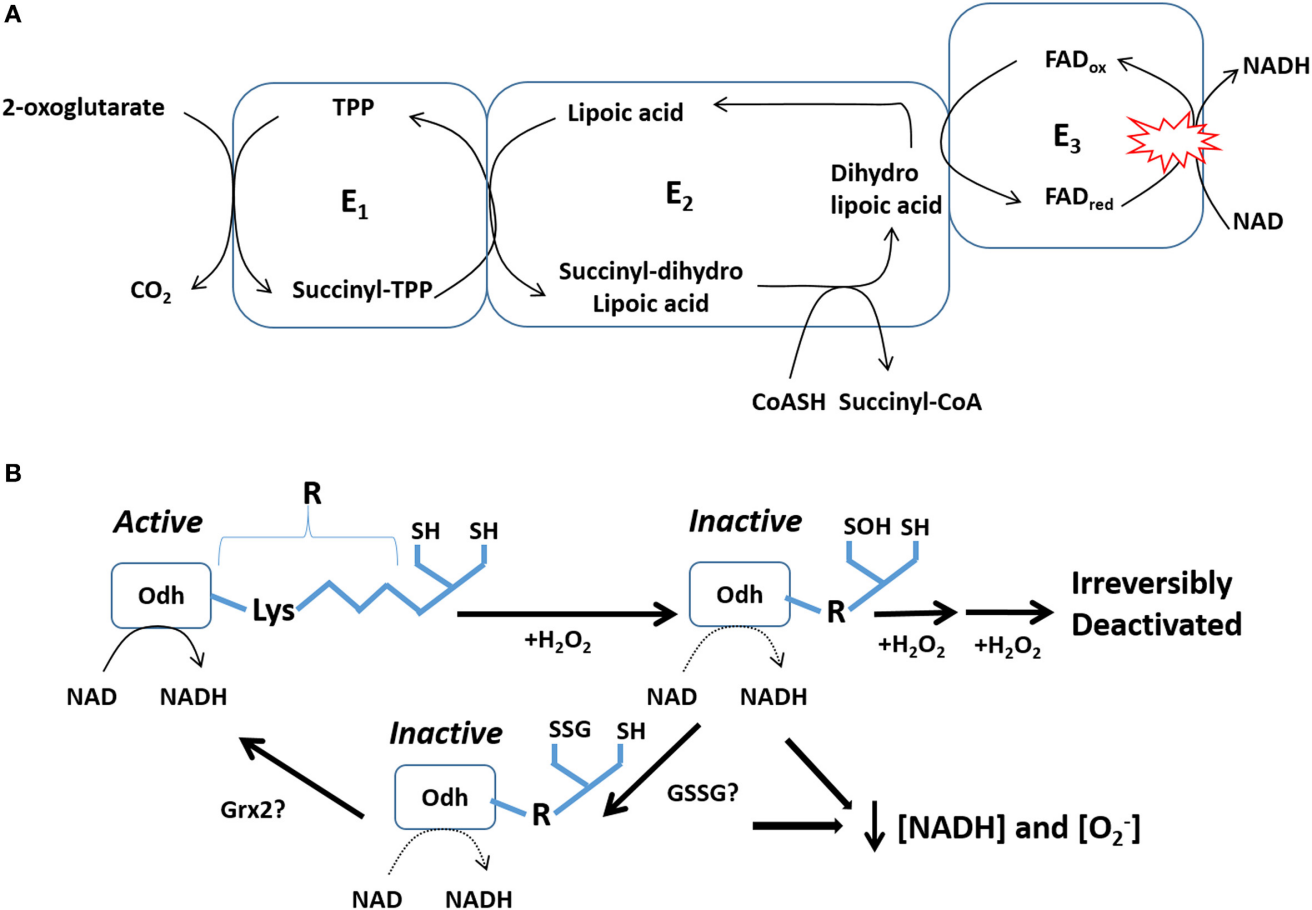
**Odh serves as an important sensor for mitochondrial O_2_•^−^/H_2_O_2_ levels. (A)** Odh-mediated conversion of 2-oxoglutarate into succinyl-CoA depends on electron transfer reaction to NAD generating NADH. Movement of electrons from 2-oxoglutarate is facilitated by lipoic acid and FAD in the E_2_ and E_3_ subunits. *Red circles*: represent the major site for O_2_•^−^/H_2_O_2_ by Odh. **(B)** Regulation of Odh by reversible S-glutathionylation and how this mechanism could hypothetically modulate the emission of O_2_•^−^/H_2_O_2_ from this enzyme complex. A small increase in mitochondrial H_2_O_2_ oxidizes vicinal thiols (SH) in Odh to sulfenic acid (SOH) which deactivates Odh. To avoid irreversible oxidation, SOH is conjugated to glutathione which can be subsequently removed, possibly by Grx2, to reactivate Odh.

Pdh and Odh are multienzyme complexes that utilize various cofactors and coenzymes including thiamine pyrophosphate (TPP, E_1_), lipoic acid (E_2_), coenzyme A (CoASH, E_2_substrate), FAD (E_3_), and NAD (E_3_ substrate) to catalyze the conversion of pyruvate (Pdh) or 2-oxoglutarate into acetyl-CoA or succinyl-CoA, respectively (McLain et al., 2011). The overall catalytic cycle for both enzymes begins with the formation of a covalent bond between TPP and substrate and the elimination of CO_2_ (Figure 2). The carbanionic substrate is then transferred to lipoic acid which then reacts with CoASH to generate either acetyl-CoA or succinyl-CoA. The reduced dihydrolipoate residue is then oxidized by FAD and the liberated electrons are passed to NAD yielding NADH. Pdh and Odh serve as major entry points into the Krebs cycle and are thus important control points for the flux of metabolites into and out of the Krebs cycle (Humphries and Szweda, 1998).

Pdh and Odh are highly sensitive to redox regulation. In particular, the lipoate residue in the E_2_ subunit is sensitive to oxidative deactivation by H_2_O_2_. This effectively limits Pdh and Odh activity prompting the accumulation of pyruvate or 2-oxoglutarate which can quench ROS albeit with very slow kinetics (Mailloux et al., 2007, 2013a). This also limits NADH production which aids in preventing the further genesis of O_2_•^−^/H_2_O_2_ by either enzyme or the respiratory chain. If H_2_O_2_ is too concentrated the E_2_ subunit can be irreversibly deactivated by overoxidation. Low μM amounts of H_2_O_2_ can rapidly oxidize the thiol residues on lipoic acid to SOH and deactivate Odh (Figure 2) (Applegate et al., 2008). SOH can then undergo further oxidation by H_2_O_2_ generating sulfinic (SO_2_H) and sulfonic acid (SO_3_H) which irreversibly deactivates the enzyme complex. This could be detrimental since it would disable the flux of metabolites through the Krebs cycle. Irreversible deactivation is prevented by S-glutathionylation of SOH groups. In a series of publications, Sweda et al. firmly established that the vicinal lipoate thiols in Odh can be rapidly oxidized by μM amounts of H_2_O_2_ followed by rapid S-glutathionylation of SOH groups in rat heart mitochondria (Nulton-Persson et al., 2003; Applegate et al., 2008; McLain et al., 2013). In addition, the same group showed that S-glutathionylation can be reversed by exogenous Grx1 and that calcium enhances the probably of S-glutathionylation (Figure 2) (Applegate et al., 2008). These seminal findings illustrate that Odh is a major focal point for rapid regulation by fluctuations in redox environment (Figure 2). Indeed, a burst in H_2_O_2_ production in mitochondria may be required to feedback and deactivate Odh and possibly Pdh to curtail further H_2_O_2_ production by either Odh and Pdh or the respiratory complexes (Figure 2) (Young et al., 2000; Green et al., 2003). S-glutathionylation prevents further deactivation and ensures that Odh can be rapidly reactivated possibly by Grx2.

The vicinal lipoate thiols on the E_2_ subunit of Odh can be subjected to a range of oxidative modifications. Odh is deactivated by H_2_O_2_-induced oxidative stress in nerve terminals which has strong implications for Parkinson's disease (Tretter and Adam-Vizi, 1999; Kumar et al., 2003). Thiolates and SOH groups can also be targeted for alkylation. For instance, lipid damage following oxidative stress generates 4-hydroxy-2-nonenal (HNE), a highly reactive electrophile that can rapidly conjugate to reactive thiols and SOH groups (Forman et al., 2008). Odh is a target for deactivation by HNE (Humphries and Szweda, 1998). Treatment of cardiac mitochondria with HNE results in a rapid decrease in energy metabolism and deactivation of Odh and formation of HNE adducts is a hallmark of cardiac and neurological diseases (Humphries and Szweda, 1998). S-glutathionylation of Odh and possibly Pdh and Bckdh prevents HNE adduct formation and/or overoxidation of lipoate groups by blocking vicinal thiols. Disabling S-glutathionylation may have strong implications for development of various neurological and cardiovascular diseases. A hallmark for heart and neurological disease is the depletion of mitochondrial glutathione pools which coincides with diminished mitochondrial respiration and metabolism (Jha et al., 2000; Mari et al., 2009). Depletion of mitochondrial glutathione would inevitably result in the loss of a key protective mechanism required to retain Odh and possibly Pdh and Bckdh activity. In fact, therapies for treating various neurological disorders are aimed at preserving mitochondrial glutathione levels (Pastore et al., 2013; Smeyne and Smeyne, 2013). Thus, Odh, Pdh, and Bckdh serve as important regulatory hubs for reversible S-glutathionylation reactions which if disabled can lead to the development of various disorders.

### Complex I; NADH:ubiquinone oxidoreductase

Complex I catalyzes the two-electron oxidation of NADH and reduction of ubiquinone to ubiquinol which is coupled to the translocation of ~4 H^+^ to the P-side of the MIM (Verkhovskaya and Bloch, 2013). Overall Complex I has 46 subunits and forms an L-shaped protein complex composed of a hydrophilic arm that protrudes into the matrix and a membrane arm that is embedded in the MIM (Mimaki et al., 2012). The hydrophilic arm consists of two modules, the N-module which contains the NADH oxidation site and FMN and the Q-module which harbors the ubiquinone binding site (Mimaki et al., 2012). In between the NADH oxidation and ubiquinone binding sites are 8–9 Fe-S clusters which link hydride transfer from NADH to FMN in the N-module to the two electron reduction of ubiquinone in the Q-module (Verkhovskaya and Bloch, 2013). Note that the ubiquinone binding site is thought to sit at the interface between the Q-module and P-module (proton pumping membrane arm) (Mimaki et al., 2012). Complex I activity is heavily regulated by allosteric and covalent mechanisms which converge on both the N-module and Q-module. The N-module consists of three main nuclear encoded proteins Ndufv1, Ndufv2, and Ndufs1 (Mimaki et al., 2012). The Q-module consists mainly of nuclear encoded proteins Ndufs3, Ndufs8, Ndufs2, and Ndufs7 (Mimaki et al., 2012). The P-module is composed of highly hydrophobic proteins encoded by the mitochondrial genome. Several subunits in the N-module have been identified as key sites for regulation by S-glutathionylation. Specifically Ndusf1 (~75 kDa) and Ndufv1 (~51 kDa) have been identified as major S-glutathionylation targets (Hurd et al., 2008; Chen et al., 2010; Mailloux et al., 2014). S-glutathionylation of either protein results in a decrease in Complex I activity. Complex I has been shown to be modulated by S-glutathionylation in a number of tissues including cardiac, liver, and lens epithelia with Ndusf1 and Ndufv1 serving as chief targets (Hurd et al., 2008; Passarelli et al., 2010; Wu et al., 2011; Mailloux et al., 2013c). The accessory subunit Ndufa11, which plays a role in Complex I assembly and stability, has also been shown to be S-glutathionylated and displays altered S-glutathionylation patterns in isolated mouse hearts subjected to ischemia-reperfusion (Kumar et al., 2013).

Some of the most intriguing results concerning S-glutathionylation of Complex I in health and disease come from Ndusf1. Ndusf1 can be S-glutathionylated on Cys^531^ and Cys^704^ in bovine heart mitochondria which lowers its activity (Hurd et al., 2008). Cys^531^ and Cys^704^ are close to the NADH binding site and thus it is probable that S-glutathionylation induces a conformational change in the N-module which diminishes NADH oxidation (Hurd et al., 2008). The most intriguing aspect of Ndusf1 is that it is reversibly S-glutathionylated by Grx2 (Beer et al., 2004; Mailloux et al., 2014). It has now been shown in bovine and mouse heart mitochondria that Grx2 can deglutathionylate Complex I. Moreover, Beer et al. was able to show that Grx2 can also S-glutathionylate Complex I (Beer et al., 2004). The capacity of Grx2 to catalyze either deglutathionylation or S-glutathionylation depends on the redox state of the 2GSH/GSSG pair. A more oxidized ratio activates Grx2 glutathionylase activity whereas a more reduced ratio has the opposite effect. In addition Mailloux et al. was able to show that Grx2 knockout (Grx2^−/−^) inhibits Complex I activity by S-glutathionylation of Ndusf1 which can be reversed by (1) restoring the redox environment through GSH supplementation and (2) addition of exogenous Grx1 (Mailloux et al., 2014).

As described above Grx2 is required to reverse S-glutathionylation of Complex I. In addition, Complex I can be easily S-glutathionylation by an increase in GSSG (Beer et al., 2004; Passarelli et al., 2010; Mailloux et al., 2014). In a recent study, Mailloux et al. provided evidence that loss of Grx2 function results in development of left ventricular hypertrophy and localized fibrosis which is associated with increased reliance on glycolysis to meet energy demands (Mailloux et al., 2014). In addition, Grx2^−/−^ mice also develop hypertension which often goes hand in hand with cardiac hypertrophy (Mailloux et al., 2014). This was associated with increased O_2_•^−^ production and inhibition of Complex I activity in cardiac tissue and a ~50% decrease in mitochondrial ATP production (Mailloux et al., 2014). The massive decline in mitochondrial ATP production is highly significant for cardiac tissue considering the contracting heart can turnover ~30 kg of ATP a day and relies on mitochondria to fulfill ~90% of its energy demands (Rosca and Hoppel, 2010). The decrease in mitochondrial ATP production was associated with decreased Complex I activity which could be easily reversed by restoration of reductive environment in mitochondria with DTT or GSH (Mailloux et al., 2014). The observation that restoration of the redox environment recovers ATP production and Complex I activity indicates the possibility that pharmacological agents can be designed and targeted to mitochondria to ameliorate deregulated S-glutathionylation reactions. These results are consistent with other studies where overexpression of Grx2 protects mouse heart mitochondria from doxorubicin-induced cardiac injury by maintaining State 3 respiration (Diotte et al., 2009). Further, Grx2 overexpression protects from apoptosis and prevents cytochrome C release by maintaining Complex I activity (Enoksson et al., 2005). Thus, S-glutathionylation reactions play a central role in modulating mitochondrial ATP in cardiac tissue, most likely in response to changing energy demands and ROS levels, and deregulation of these reactions can lead to development of heart disease.

Another intriguing aspect for modulation of Complex I activity through thiol modification is the active/deactive (A/D) transition. Complex I can cycle between A and D-forms which is dependent on NADH and ubiquinone (Grivennikova et al., 2001). What is interesting here is that transition to D-form results in exposure of a conserved cysteine residue which can be readily modified by thiol modifying reagents N-ethylmaleimide or iodoacetamide (Babot et al., 2014). It has been established that Cys^39^ on Complex I subunit ND3, which forms part of the P-module, becomes exposed upon A to D transition and can then be modified by alkylating agents. Notably Cys^39^ of ND3 makes contact with the solvent inaccessible ubiquinone binding site which sits at the interface between the hydrophilic and hydrophobic arm of Complex I (Babot et al., 2014). Thus, during two-electron oxidation of NADH the ubiquinone binding site is closed ensuring electron transfer and reduction of ubiquinone can proceed. When substrate abundance is low, ND3 undergoes a conformational change exposing Cys^39^ making it amenable for modification resulting in Complex I deactivation. Of note Cys^39^ of ND3 subunit can undergo S-nitrosylation which was shown to protect cardiac tissue from ischaemia-reperfusion and reduce myocardial infarct size (Chouchani et al., 2013). Dröse et al. proposed that Cys^39^ on ND3 may also serve as an important site for regulation of Complex I activity by redox modifications like S-glutathionylation (Drose et al., 2014).

**Figure 3:**
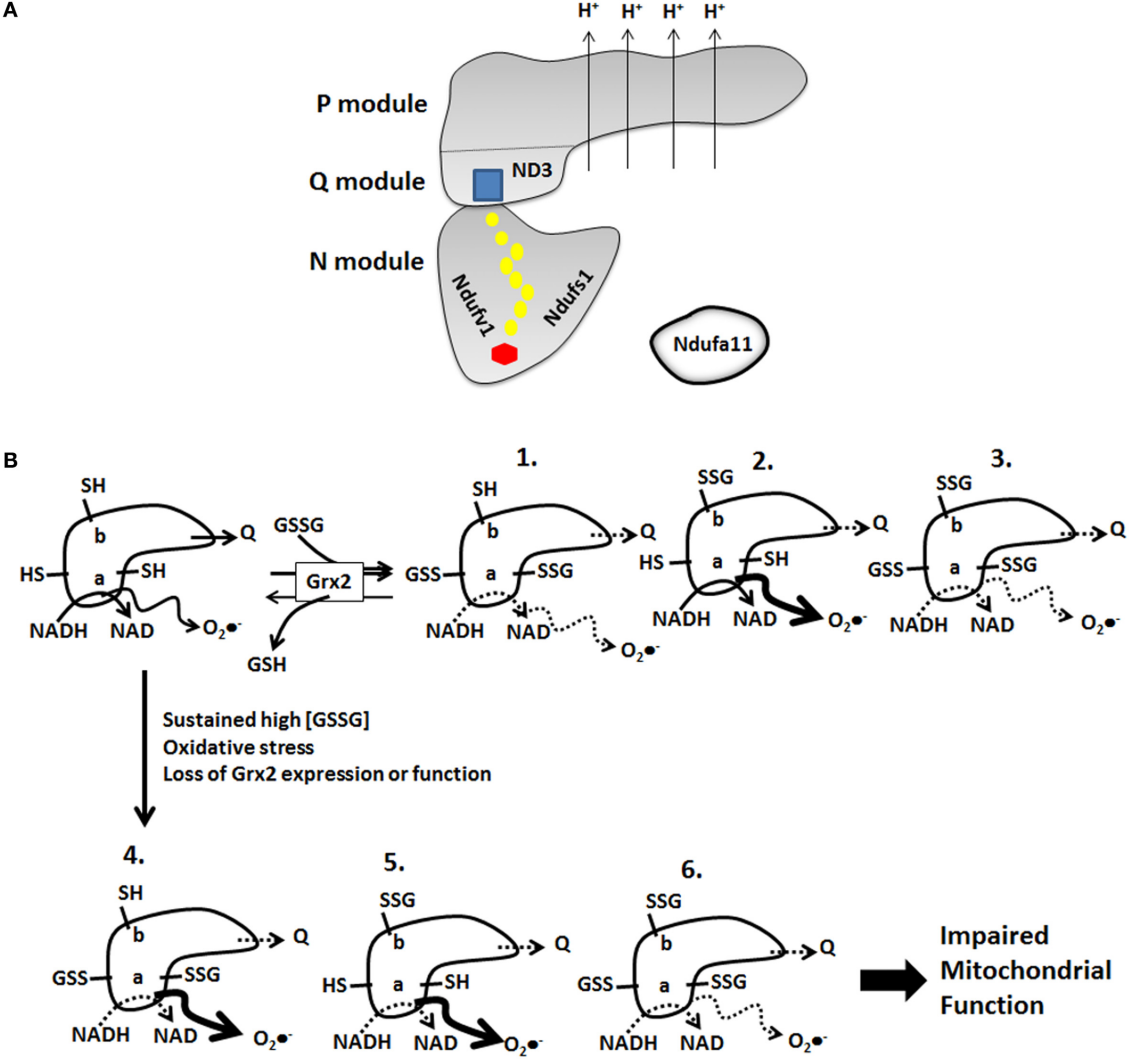
**Complex I harbors multiple S-glutathionylation sites which may serve as a “cysteine code” required to modulate its activity under various conditions. (A)** Representative diagram illustrating the basic structure of Complex I and the location of the various modules, prosthetic groups, and proton pumping domains. Subunits that can be S-glutathionylated are highlighted. Hexagon; FMN, circles; Fe-S clusters, square; ubiquinone binding site. **(B)** Complex I cysteine code. Hypothetical schematic depicting the different possible S-glutathionylation states that can be adopted by Complex I under physiological (regulatory) and pathological conditions. Note that S-glutathionylation of the different subunits in different combinations ultimately lowers Complex I activity but duration and location of S-glutathionylation can have variable effects on O_2_•^−^ emission. “a” corresponds to Ndufs1 or Ndufv1 and “b” corresponds to ND3. Note that accessory protein Ndufa11 has been excluded for clarity but may also be differentially S-glutathionylated in response to various physiological and pathological stimuli.

Studies have found that Complex I S-glutathionylation can increase and decrease O_2_•^−^ formation. Hurd et al. found that S-glutathionylation of Ndusf1 diminishes O_2_•^−^ formation by the Complex which is most likely due to blocking hydride transfer from NADH to FMN and prevention of the formation of FMN semiradicals during 1 electron transfer to Fe-S in the Complex (Hurd et al., 2008). However, the same group and others have shown S-glutathionylation of Complex I can increase O_2_•^−^ production (Taylor et al., 2003; Mailloux et al., 2014). There are two reasonable explanations for this discrepancy. The first is that S-glutathionylation may initially prevent O_2_•^−^ production by Complex I but chronic/prolonged S-glutathionylation may have the opposite effect (e.g., increase O_2_•^−^ production). This is especially relevant for cardiac tissue considering that fatty acid oxidation (FAO) accounts for ~60–90% of the ATP produced by mitochondria. Prolonged inactivation of Complex I can amplify FAO-mediated O_2_•^−^ production from a number of sites in the ETC and from Odh and Pdh due to an increase in NADH/NAD (Figure 3). In terms of short term regulation S-glutathionylation may diminish O_2_•^−^ production following a burst in O_2_•^−^/H_2_O_2_ production by mitochondria (Figure 3). This leads to an increase in GSSG and S-glutathionylation of Complex I decreasing its activity and O_2_•^−^ production. Grx2 is activated by the burst of O_2_•^−^ production which subsequently deglutathionylates and reactivates Complex I (Figure 3). This hypothetical mechanism is anticipated to be very rapid mode of regulation required to adjust O_2_•^−^ production and ROS emission by mitochondria in response changes in redox environment (Figure 3). It also most likely plays a central role in controlling ROS signaling from mitochondria to the rest of the cell. Depending on the energy demands of cardiac tissue this mechanism may also be required to control ATP output. However, disabling Grx2 or oxidative stress can induce prolonged Complex I S-glutathionylation which could eventually drastically amplify mitochondrial ROS emission (Figure 3). The second explanation for this discrepancy is Complex I harbors multiple S-glutathionylation sites which may affect whether or not O_2_•^−^ production is increased or decreased (Figure 3). Indeed, both Ndufs1 and Nduv1 form part of the NADH binding site and S-glutathionylation of either protein or both may actively prevent the two-electron reduction of FMN by NADH—however, prolonged S-glutathionylation may result in electron backflow from a reduced quinone pool and production of O_2_•^−^ by FMN (Figure 3). Ndufa11 S-glutathionylation may have a similar effect regulating Complex I assembly thereby modulating respiratory complex content in mitochondria which would affect ROS production. In addition, the observation that Ndufa11 is S-glutathionylated would indicate that the mitochondrial redox environment plays an intricate role in respiratory complex assembly and possibly even respirasome assembly. Concerning ND3, although it has a modifiable cysteine residue it remains unknown if it is S-glutathionylated. However, it is tempting to speculate that it is S-glutathionylated and that it also influences O_2_•^−^ production by Complex I. In this particular scenario ND3 S-glutathionylation and prolonged deactivation of Complex I may actually increase O_2_•^−^ formation (Figure 3). S-glutathionylation of ND3 may prevent ubiquinone binding prompting a build-up in FMN radical and a subsequent increase in O_2_•^−^ formation (Figure 3). It would be important to decipher if S-glutathionylation of any of these proteins, perhaps using Cys substitution models and knockouts, enhances or diminishes mitochondrial O_2_•^−^production. Also it would be important to determine the circumstances under which each subunit is S-glutathionylated and if all subunits can be S-glutathionylated simultaneously and the impact different S-glutathionylation states has on Complex I function. Thus, it remains unknown if all subunits (Ndufs1, Ndufv1, Ndufa11, and ND3) can be S-glutathionylated simultaneously or if these subunits are differentially S-glutathionylated under different conditions, either physiological (e.g., regulation) or under oxidative stress/pathological conditions. As shown in Figure 3, we have generated six different hypothetical models illustrating what may or may not happen when Complex I is S-glutathionylated on these different subunits in different combinations and what may occur under (1) regulatory and (2) pathological conditions. We propose that Complex I actually harbors an S-glutathionylation cysteine code that is required to fine tune cardiac metabolism in response to various stimuli and that disruption of this signaling platform is detrimental to heart physiology.

### Complex II; succinate dehydrogenase

**Figure 4:**
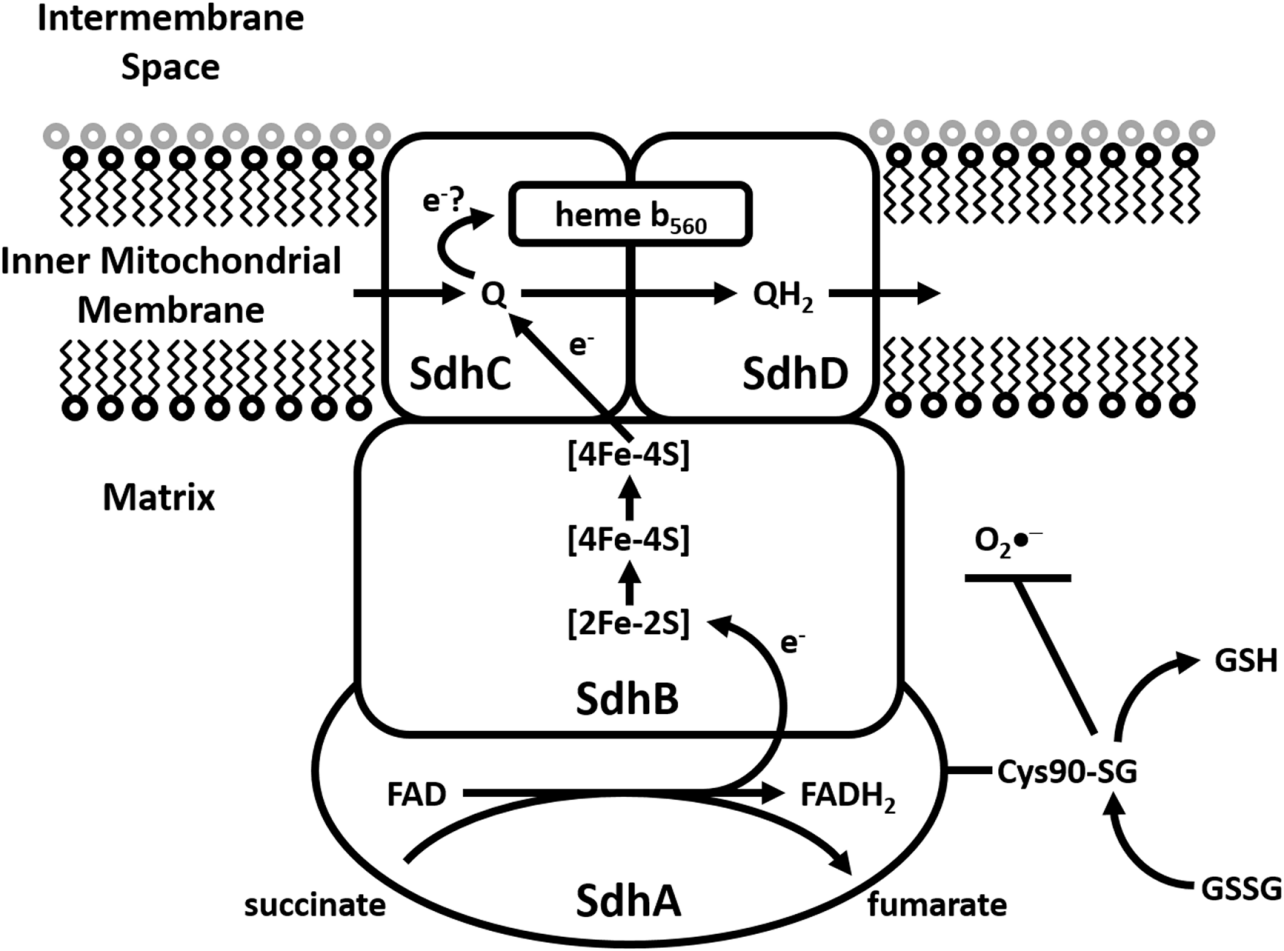
**Modulation of Sdh by S-glutathionylation**. Representative diagram illustrating electron flow from succinate to ubiquinone in Sdh with subsequent production of fumarate. The S-glutathionylation of Cys^90^ on SdhA subunit is required to maintain succinate oxidation activity and diminish O_2_•^−^ production. Deregulation of SdhA S-glutathionylation results in diminished activity and increased mitochondrial ROS production which is associated with ischemic damage of cardiac tissue.

Succinate dehydrogenase [(Sdh) succinate-coenzyme Q reductase or succinate-ubiquinone oxidoreductase] is a multienzyme complex bound to the inner membrane of the mitochondria and many bacterial cells (Figure 4). It has multiple roles, participating in both the citric acid cycle as well as the ETC. Sdh catalyzes the oxidation of succinate to fumarate, with the corresponding reduction of ubiquinone to ubiquinol. Sdh is comprised of four protein subunits (SdhA, SdhB, SdhC, and SdhD) and a heme center. SdhA (a flavoprotein which converts FAD to FADH_2_) and SdhB (containing three iron-sulfur clusters; [2Fe-2S], [4Fe-4S], and [3Fe-4S]) are hydrophilic and occur within the mitochondrial matrix. SdhC and SdhD are hydrophobic and occur within the inner leaflet of the MIM, binding a heme b_560_ molecule in between them. The oxidation of succinate to fumarate, with the corresponding reduction of FAD to FADH_2_ occurs in SdhA. Electrons derived from FAD tunnel through the iron-sulfur clusters of SdhB (from [2Fe-2S] to [4Fe-4S] to [3Fe-4S]) to a waiting ubiquinone in the active site of SdhC. The heme prosthetic group is suggested to be an electron sink, with electrons tunneling back-and-forth between ubiquinone and heme b_560_. Thus, the heme may prevent electrons escaping to molecular oxygen and forming O_2_•^−^. Indeed, mounting evidence has suggested that Sdh can be a significant source of ROS. Studies show that ROS production by inhibition of Sdh occurs both indirectly and directly. In terms of the former this occurs through inhibition of the ubiquinone binding site (atpenin A5, TTFA) or the succinate binding site (malonate) and the latter via autooxidation of flavin (Zhang et al., 1998).

Removal of Sdh function in mice has been shown to be embryonic lethal (Piruat et al., 2004). Mutations in the SdhA subunit can lead to Leigh syndrome, mitochondrial encephalopathy, and optic atrophy (Jain-Ghai et al., 2013). Mutations in SdhB, SdhC and SdhD subunits lead to tumorogenesis in chromaffin cells, as well as in the head and neck regions, resulting in hereditary paraganglioma and hereditary pheochromocytoma (Bardella et al., 2011; Hoekstra and Bayley, 2013). These mutations also lead to increased O_2_•^−^ production and decreased lifespan (Slane et al., 2006). Mutations in SdhC, in particular, may lead to alteration of the binding site for ubiquinone, causing a rerouting of the electron flow from ubiquinone to molecular oxygen. Chronic O_2_•^−^ production, dismutated to H_2_O_2_, in these mutants results in localized genomic instability and other phenotypic changes related to the development of cancer and cellular aging. Whereas tumors resulting from SdhB mutation tend to be malignant, tumors resulting from SdhC and SdhD mutations tend to be benign.

Several studies have conclusively shown that S-glutathionylation is required to modulate Sdh and that perturbations of Sdh S-glutathionylation are associated with ischemia-reperfusion injury in cardiac tissue (Chen et al., 2007). Intriguingly Sdh is actually deglutathionylated following ischemia-reperfusion injury which diminishes succinate oxidation and enhances O_2_•^−^ by Complex II (Figure 4) (Chen et al., 2007). It would appear that in this case S-glutathionylation, specifically of Cys^90^ on SdhA subunit, is required to maintain activity. It has been proposed that S-glutathionylation of Sdh is actually required to protect the enzyme from oxidative deactivation possibly via a mechanism similar to Odh. Indeed, it was also shown that S-glutathionylation of Sdh is required to protect the enzyme complex from oxidative deactivation. Specifically this is Cys^90^ of the SdhA subunit has been shown to be S-glutathionylated using purified SdhA and liquid chromatography/tandem mass spectrometry. *In vitro* S-glutathionylation of Sdh has been shown to protect the enzyme from thiyl radical formation induced by O_2_•^−^ (Chen et al., 2007) as well as against peroxynitrite-mediated tyrosine nitration and impairment of the enzyme's protein-protein interaction with Complex III (Chen et al., 2008). Such damaging tyrosine nitration of SdhA has been found to occur in the post-ischemic myocardium (Chen et al., 2007). Cys^90^ of SdhA is persistently glutathionylated and thus continuous modification of this subunit is considered essential to the optimal functioning of this enzyme.

### Complex V; F_0_F_1_ ATP synthase

**Figure 5:**
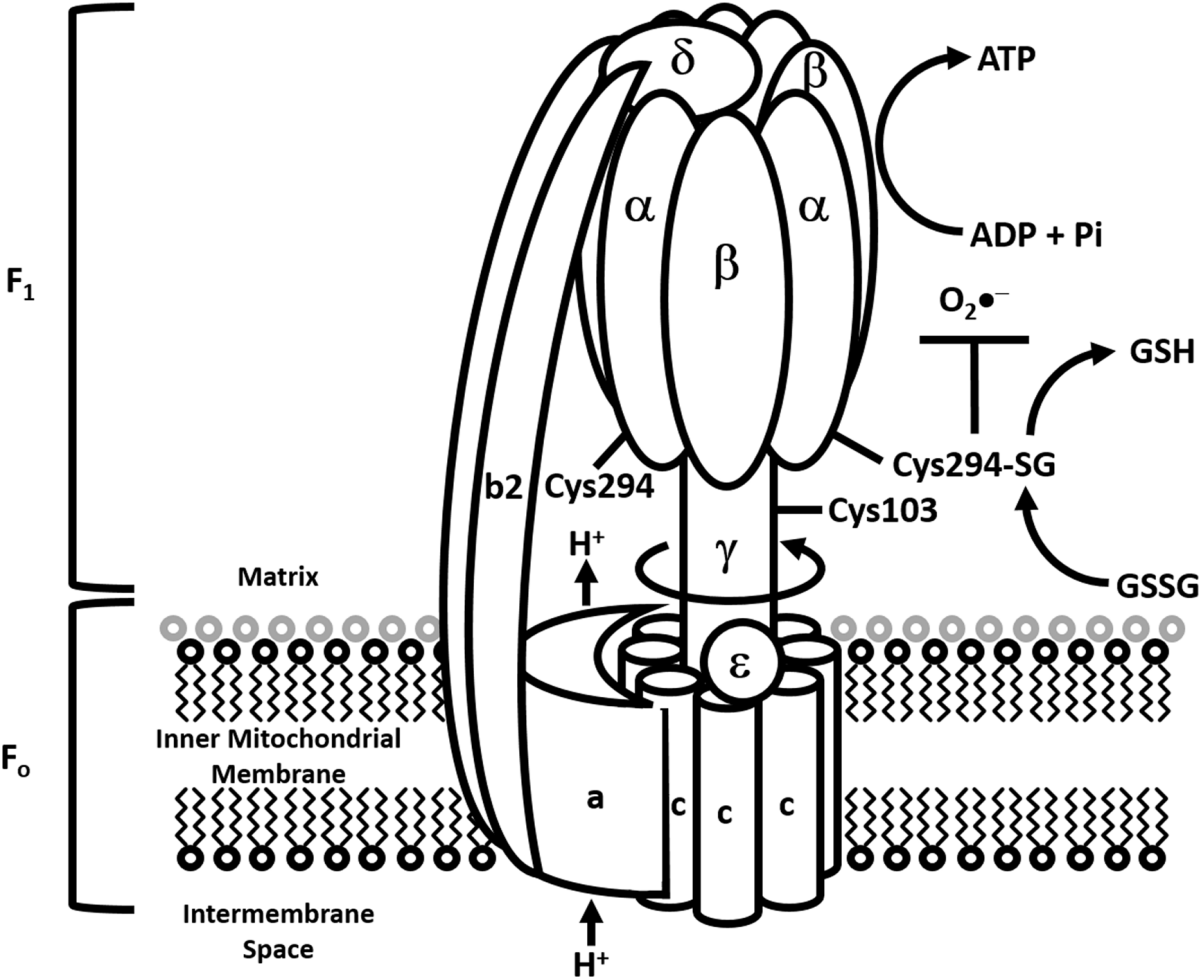
**Modulation of ATP synthase by S-glutathionylation**. Diagrammatic representation of the ATP synthase complex and how it couples proton transport to the synthesis of ATP. S-glutathionylation of α-subunit in the F_1_ portion of the complex is required to modulate ATP synthesis and curtail the genesis of mitochondrial O_2_•^−^ production. However, prolonged S-glutathionylation is associated with chronic decreases in mitochondrial ATP production and development of heart disease.

ATP synthase is an enzyme that synthesizes the energy currency of the cell, adenosine triphosphate (ATP) (Figure 5). Complex V has a hydrophobic F_o_ portion present within the inner membrane of the mitochondria and a hydrophilic F_1_ portion present within the matrix of the mitochondria. The F_0_ portion of the enzyme contains subunits A, B, and C while the F_1_ portion contains subunits alpha (α), beta (β), gamma (γ), delta (δ), and epsilon (ε). The pmf across the inner mitochondrial membrane, generated by the ETC, drives the passage of protons through the membrane via the F_o_ region of ATP synthase. A ring of 8-15 C subunits (depending upon the species), and the ε subunit, rotates as protons pass through the membrane. This ring of C subunits is tightly attached to a central stalk, consisting of the γ subunits, which rotates within the fixed α_3_β_3_ subunits of F_1_. Protons pass through channels in the A subunit, which rests against the C subunit ring, from the mitochondrial intermembrane space to the matrix as the C subunit ring turns. F_0_ is also connected to F_1_ by a peripheral stalk comprised of two B subunits and one δ subunit. The rotation of the γ subunit within the α_3_β_3_ subunits of F_1_ causes conformational changes in the ADP + Pi binding site of the β subunits, resulting in the conversion of ADP to ATP. After one complete rotation of F_1_, ATP is released, allowing another ADP + Pi to bind.

The generation of ROS by mitochondria is highly dependent upon mitochondrial membrane potential (ΔΨm) and the amount of ROS produced can either trigger cellular proliferation (low ROS) or apoptosis (high ROS) (Martinez-Reyes and Cuezva, 2014). Inhibition of ATP synthase by 1,4-benzodiazepine-derivative Bz-423 induces mROS production and signals apoptosis. This is the result of an increase in ΔΨm and subsequent superoxide production from the mitochondrial ETC (Martinez-Reyes and Cuezva, 2014). ATPase Inhibitory Factor 1 (IF1) is a low molecular weight (~10 kDa) mitochondrial protein which acts as a natural inhibitor of ATP synthase, but does not interfere with its coupling activity (Cabezon et al., 2000). IF1 is activated under low pH conditions (which occurs with a change in ΔΨm) and binds to the α, β, and γ subunits of ATP synthase. Binding of IF1 inhibits the conformational changes required for ATP binding to the αβ sites of F1 and thus it disrupts the catalytic site of ATP synthase (Campanella et al., 2008). Inhibition of the ATP synthase increases mitochondrial ΔΨm and increased superoxide production from the mitochondria, activating proliferation and survival pathways within the nucleus of the cell and promoting tumorigenesis and metastasis (Formentini et al., 2012). IF1 is highly overexpressed in tumor cells which also shifts the cell toward a more glycolytic phenotype.

In the heart, the α subunit of ATP synthase is glutathionylated during dyssynchronous heart failure (DHF) and this can be induced by GSSG in a dose-dependent manner. Interestingly, the extent to which the α subunit of ATP synthase is glutathionylated is partially normalized in cardiac resynchronization therapy (CRT), providing evidence that this effective treatment for heart failure patients also has beneficial effects in terms of oxidative post-translational modification of mitochondrial proteins (Wang et al., 2011). The α subunit of ATP synthase provides a redox switch for the enzyme's function under normal conditions and conditions of oxidative stress (Wang et al., 2011) (Figure 5). Cys^294^ of the α subunit can be oxidized to Cys^294^-SOH with superoxide exposure or Cys^294^-SNO with nitric oxide exposure (with increasing oxidative stress). These modifications can be further oxidized to form a disulfide bridge with either Cys^294^ of an adjacent α subunit, Cys^103^ of the γ subunit of ATP synthase or glutathione (S-glutathionylation) (Wang et al., 2011). All three disulfide bridges cause a substantial decrease in ATP synthase activity, leading to mitochondrial dysfunction. Antioxidant systems that reverse disulfide bond formation would enhance ATP synthase function under these conditions. Reversible S-glutathionylation of Cys^294^ of the α subunit of ATP synthase, although initially detrimental to the enzymes activity, would protect it from the irreversible modification of sulfenic to sulfinic to sulfonic acid formation. Other studies have shown that different Cys residues on the α subunit of ATP synthase to be S-glutathionylated during oxidative stress (upon H_2_O_2_ exposure of isolated brain mitochondria) (Garcia et al., 2010). A previous study identified one of the amino acid residues glutathionylated to be Cys^163^ (West et al., 2006).

## Conclusion and perspectives

Mitochondria are masters of energy conservation. In mitochondria, electron transfer potentials are coupled to the genesis of transmembrane protonic forces, a form of Gibbs free energy that can be readily utilized for the synthesis of ATP. It is through the process of oxidative phosphorylation that mitochondria were able to aid in the development of evolutionary complexity and the inevitable “ascent of man” (Wallace, 2010). Electron transfer processes in mitochondria can also lead to the production of ROS. Once viewed as unfortunate waste products of respiration it is now well known that ROS serve as an important means for mitochondria to regulate its own functions or communicate with the rest of the cell. However, mitochondria must strike a delicate balance when using ROS as a signaling molecule since over production can induce cell damage and death. Indeed, mitochondrial dysfunction which is typically characterized by an ATP production deficiency and/or over production of ROS can lead to cell death and development of disease. In the present article, we discussed emerging evidence and hypotheses around the role of S-glutathionylation reactions in the control of mitochondrial metabolism and function in response to fluctuations in redox environment. In addition, we provided a framework for how disruption of S-glutathionylation signaling cascades can lead to mitochondrial dysfunction and development of disease. S-glutathionylation reactions, mediated by Grx2, play a central role in regulating mitochondrial ATP production and ROS emission in response to local and robust changes in redox environment, in particular fluctuations in H_2_O_2_ and 2GSH/GSSG. Although only a handful of Grx2 targets have been identified there are a number of mitochondrial proteins, which fulfill a multitude of functions that are known to undergo S-glutathionylation. It is important to ascertain if these mitochondrial proteins can also be targeted by Grx2 and the circumstances under which these proteins are reversibly S-glutathionylated in response to redox environment fluctuations.

### Conflict of interest statement

The authors declare that the research was conducted in the absence of any commercial or financial relationships that could be construed as a potential conflict of interest.
